# Improvement in Brazilian barley breeding: Changes in developmental phases and ecophysiological traits

**DOI:** 10.3389/fpls.2022.1032243

**Published:** 2022-11-25

**Authors:** Osmar Rodrigues, Euclydes Minella, Edson Roberto Costenaro, Silvia Scariotto, José Abramo Marchese

**Affiliations:** ^1^ Brazilian Agricultural Research Corporation, Embrapa Wheat, Passo Fundo, RS, Brazil; ^2^ Department of Agronomy, Federal University of Technology – Parana (UTFPR), Pato Branco, PR, Brazil

**Keywords:** developmental phases, intercepted radiation, LAI, phyllochron, tillering

## Abstract

Despite recognizing the importance of genetic improvement in the production of barley grains, little information is available on the contribution of phenological development to the genetic improvement of Brazilian barley. Field experiments were carried out between 2011 to 2013, in the absence of biotic and abiotic stresses and with preventive lodging control. Five two-rowed spring barley cultivars, released between 1968 and 2008, were evaluated. Although there was no significant association in the cycle length (Emergence - Anthesis) of the cultivars with the year of release, the genetic improvement increased the proportion of the Doble ridge - Maximum number of spikelet primordia/Maximum number of spikelet primordia - anthesis period to the total time to anthesis. The period between DR-MNP was increased in modern cultivars, to the detriment of the Doble ridge - Maximum number of spikelet primordia period. However, the duration of the period between emergences to the double ridge (vegetative phase) was not altered in the analyzed period. Barley breeding in Brazil did not change the total number of leaves on the main stem but caused an increase in the number of leaves earlier in the development, favoring the high level of tillering. The leaf architecture of modern barley was altered towards a more vertical inclination (erectophilic canopy), allowing the penetration of photosynthetically active radiation into the crop canopy.

## Introduction

Barley (*Hordeum vulgare* L.) is a winter cereal that occupies the forth position, in order of economic importance, in the world. The forecast for global barley production 2022/2023 was increased by 2.5 million tonnes, and now stands at 147.9 million tonnes, 1.7% higher on an annual basis (FAO, 2022). The forecast for Brazilian barley production in 2022 is 540,700 tonnes, with an increase of 23.8% compared to 2021 (IBGE, 2022). In Brazil, barley breeding began in the middle of the 20th century, focusing on resistance to diseases, resistance to lodging, tolerance to different environmental stresses, malt quality, and grain yield (Rodrigues et al., 2020). However, the effect of genetic improvement of barley on some physiological attributes (e.g. changes in developmental phases and ecophysiological traits), has not being reported making it difficult for the scientific community to understand the performance of this crop under Brazilian growing conditions.

The phenological development of barley could be divided into three main phases: vegetative, reproductive, and grain filling. The vegetative phase begins with sowing and lasts until floral initiation, and the initiation of leaves and tillers characterizes it. The reproductive phase starts with the differentiation of the spikelet at the apex and ends with ovary pollination within the spikelets, usually coinciding with the heading stage. Further, the reproductive phase can be divided into two-sub-phases: a) initial reproductive: where the spikelet differentiation is initiated at double ridge stage (DR) until a maximum number of spikelets primordia (MNP) is reached, equivalent to terminal spikelet stages (TS) in wheat and awn primordium (AP) in Barley (Nerson et al., 1980; Alqudah and Schnurbusch, 2014), and b) late reproductive phase.

In barley, the late reproductive phase can be divided into three sub-phases (Alqudah and Schnurbusch, 2014): 1) awn primordium (AP) to tipping stage (first awns visible on main culm - Z49), 2) tipping to heading (half main culm spike emerged from flag leaf sheath - Z55) and 3) heading to anther extrusion (half of main culm spike with anthers - Z65) (Zadoks et al., 1974). In this late-reproductive phase, the ear elongation period occurs in parallel with the elongation of the main stem (Kirby, 1988; Reynolds et al., 2009), and the number of fertile flowers is set within the spike. The beginning of the floret primordia mortality phase coincided with the beginning of spike growth at its fastest rate and continued until the heading stage when stem and spikes are growing together with high competition for assimilates (Arisnabarreta and Miralles, 2008). Finally, the last phase of development (GF) begins after fertilization, when the seed endosperm accumulates dry matter and determines the final seed weight.

In recent decades, it has been demonstrated that not all phases of cereal development are equally important (Kirby and Appleyard, 1984; Landes and Porter, 1989; Slafer and Rawson, 1994; Rodrigues et al., 2007) for seed yield. These phases could be independent of each other (Miralles et al., 2000; Gonzáles et al., 2003) and be under different genetic control, being able to be manipulated without changing the total time until anthesis (Slafer and Rawson, 1994; Slafer et al., 1996; Slafer et al., 2001). The phase between the maximum number of primordia of spikelets at anthesis (MNP-ANT), characterized by intense spike growth and stem elongation, has been attributed as the most significant contribution to grain yield (Slafer and Rawson, 1994; Whitechurch et al., 2007; González et al., 2011). However, Alqudah and Schnurbusch (2014) studied sub-phases in barley within the reproductive phase and point out that the second sub-phase of the reproductive phase (AP–tipping) is the most critical period for spikelet survival.

During this final period (MNP-ANT), the rapid growth of the spike coincides with the source limitation due to the higher inter-plant competition and the maximum growth rate of the stem of each plant (Siddique et al., 1989). At this period in barley, the rapid growth of stem and spike growth compete for assimilates, generating spikelet abortion. Kernich et al. (1997) observed in barley cultivars a low variation in the number of spikelets observed at awn primordium stages but a considerable variation in spikelet death. Similarly, Garcia del Moral et al. (2002) observed that the final number of grains depends more on survival (degeneration) than on the generation of reproductive structures that could produce more grains. Evidence in several genotypes with similar spikelet degeneration suggests that this phenomenon may be under environmental control (García del Moral et al., 1991).

The increase in MNP to ANT period has different implications for the number of fertile flowers per ear in wheat and barley (Miralles et al., 2000). The number of fertile flowers on barley ears appears to be limited by the maximum number of fertile spikelets (approx. 30 spikelets ears^-1^). In wheat, a given spikelet structure provides greater potential for flower formation by the distal primordium. Therefore, MNP to ANT period appears to be critical for the survival of the spikelets (Cottrel et al., 1985; Gonzáles et al., 2003) and has been pointed out with potential utility for plant breeding in the sense of maximizing the production of barley grains (Miralles et al., 2000). In this sense, several strategies have been studied in this phase (MNP-ANT) to maximize their production.

The greater accumulation of dry matter in the spike, by increasing the duration of the phase between MNP-ANT, with the reduction in the time of the previous phases (Slafer et al., 1996; Araus et al., 2002; Abeledo et al., 2003; Reynolds et al., 2005; González et al., 2005; Miralles and Slafer, 2007 and Bancal, 2008), keeping the total time constant at anthesis, may be a promising strategy for increasing grain yield potential in barley. Several authors have demonstrated phenotypic variability in the duration of the phases in barley (Appleyard et al., 1982; Kitchen and Rasmusson, 1983; Kernich et al., 1995; Whitechurch et al., 2007 and Kernich et al., 1997), despite the similarity between cultivars in the time to anthesis.

Increasing the biomass partition at the expense of stem growth has also been proposed during this critical phase (Siddique et al., 1989; Slafer and Andrade, 1993). On the other hand, the increase of biomass in this period could be obtained by increasing the growth rate by the greater RUE (Radiation Use Efficiency) (Reynolds et al., 2005; Miralles and Slafer, 2007; Acreche and Slafer, 2009), through a better distribution of radiation in the canopy. This strategy has limitations since, during the MNP-ANT period, the canopy and the maximum radiation interception have already been reached.

Another critical aspect in the competition for carbohydrate and nitrogen reserves between stem growth and ear growth at the end of the reproductive phase (MNP-ANT) (Ellis and Kirby, 1980; Cottrell et al., 1985) is the concomitant development of tillers (García del Moral and García del Moral, 1995). Therefore the competition for reserve becomes even greater, leading to tiller mortality, which often begins after floral initiation (DR) in the main stem, reaching the lowest levels at ear emergence. Thus, as in winter cereals, the flowering period is related to differences in the number of leaves (Appleyard et al., 1982; Slafer and Rawson, 1996) and the emergence of the tillers, which is related to the appearance of leaves (Kirby et al., 1985) may be impaired. The synchrony between these processes is fundamental to improving crop ability to intercept solar radiation (Dofing and Karlsson, 1993).

Although in recent years, a large number of articles have been published showing the yield genetic gain in barley (Riggs et al., 1981; Wych and Rasmusson, 1983; Martiniello et al., 1987; Boukerrou and Rasmusson, 1990; Bulman et al., 1993; Jedel and Helm, 1994; Abeledo et al., 2003; Muñoz et al., 2006; Rodrigues et al., 2020), little information is available (Siddique et al., 1989; Calderini et al., 1995) on the effect of genetic improvement on the phenological development of barley and particularly on the appearance of leaves, tillers and the duration of phenological phases. Such information may be relevant to continue increasing the yield potential in barley. Riggs et al. (1981) found that breeding reduced time to anthesis by seven days in the UK, in association with a decrease in the final number of leaves on the main stem and a reduction in the leaf reduction rate. In the USA, Wych and Rasmusson (1983) found a slight difference in the period until anthesis. However, in a different study, this trend was not supported (Boukerrou and Rasmusson, 1990).

In Canada, modern cultivars also showed an MNP-ANT period later than older cultivars (Jedel and Helm, 1994). This improvement impact can have a repercussion on tillering, as previously observed by Riggs et al. (1981), where more modern cultivars not only produced more tillers but also showed a higher survival rate.

In Spain, a study with a similar objective pointed out that genetic improvement did not show a significant effect on the duration of the period until anthesis in the studied cultivars (Muñoz et al., 2006).

In Argentina, Miralles et al. (2000) in two-row barley showed that the relationship between the number of fertile spikelets per ear and the duration of the MNP-ANT period was presented as a linear model only in the first 55 days, with a number maximum of 30 fertile flowers/ears. Thus, in two-row barley, the opportunity to increase the number of fertile flowers in the spike by manipulating the development phase (MNP-ANT) would be more limited than in wheat. For in wheat, the structure of the spikelet is determinate and provides more potential for flower formation through the distal beginnings (Miralles et al., 2000). On the other hand, in two-row barley, unlike wheat, the ear structure is indeterminate and provides a greater spikelet formation potential. Thus, the duration of the DR-MNP period in two-row barley was expected to have a greater impact on the increase in fertile flowers by the greater number of spikelets per spike than the MNP-ANT period. On the other hand, Abeledo et al. (2003) availed the improvement of barley in the period between 1944 to 1998 and observed that neither time to heading nor time to maturity were systematically modified by breeding. However, the partitioning of the developmental time was modified. The ratio of the duration jointing-heading to the sowing-jointing period was increased with the year of release of the cultivars. In this study, as the final number of leaves was not different between cultivars, the high biomass produced by modern cultivars was attributed to the greater interception of radiation by the faster tillering (Abeledo et al., 2003 and Abeledo et al., 2004).

Borrás et al. (2009), studied the effect of phase duration before and after joining (which includes leaf and spikelet onset, LS, and stem elongation, SE) on developmental characteristics that affect other aspects of the generation of production, such as phyllochron and tillering, observed that changes in LS duration did not necessarily imply concomitant changes in these characteristics that could be important for early expansion of the crop canopy. Thus, the possible disadvantages to the culture’s canopy structure, associated with the increased duration of stem elongation at the expense of developmental stages before MNP, do not seem to exist.

Evaluation of the effect of genetic improvement on phenological development (barley phasic development), can help us to identify characteristics of potential value for future crosses, contributing to the increase of the production potential of the culture. In addition, little is known about the implications of changes in the duration of LS and SE upon other aspects of yield generation. Thus, the objective of the present work was to analyze the effect of barley breeding on the duration of barley development phases, tillering, phyllochron, leaf area index, interception of radiation, and the final number of leaves associated with the increase in grain yield.

## Material and methods

### General conditions and experimental design

The study was conducted at the National Wheat Research Center at Passo Fundo (28°15’ S, 52°24’ W, 687 m), RS, Brazil. Field trials were carried out in 2011, 2012, and 2013, using five two-row spring barleys. The cultivars, which represented the breeding period from 1968 to 2008, were (with their year of release and genealogy between brackets): FM 404 (1968-Selection/alpha), FM 434 (1977-Quinn/Malteria Heda//FM 424), BR 2 (1990-FM 424/TR 206), BRS 195 (2000-Defra/BR 2) and BRS Elis (2008-BRS 195/Scarlett). These cultivars were chosen to represent each of the decades based on the significant participation in the total barley area harvested in southern Brazil and were previously analyzed by Rodrigues et al. (2020) regarding genetic gain for yield.

The experiments were arranged in a complete randomized block design with four replications. The plots consisted of 12 rows, 0.20 m apart and 6 m long. The sowing dates were 17 June in 2011, 6 June in 2012, and 2013 at a rate of 400 seeds/m^2^. Nine days after seedling emergence plots were thinned to 300 plants/m^2^ to reach the recomended comercial density. The fertilizer used was 250, 300, and 300 kg/ha (NPK 5-20-25), incorporated before sowing in 2011, 2012, and 2013, respectively. In 2011 it was applied 32 kg/ha of N at the double ridge and awn primordium stages as topdressing. In 2012 and 2013, 30 kg/ha of N was applied at the same stages.

The experiments were irrigated, and weeds were controlled periodically. Diseases and insects were controlled by spraying fungicides, and insecticides and nets were installed to prevent lodging.

The data were subjected to analysis of variance and differences among treatments determined. The degree of association between the studied variables was estimated using linear and quadratic regression models. The ExpDes package was used to analysis of statistical data (Ferreira et al., 2018), and the ggplot2 package was used to data visualization (Wickham, 2016).

### Measurements

The thermal time from emergence to physiological maturity (Z92, yellow spike - Zadoks et al., 1974), was determined (**Table 1**). The time for each stage was recorded when at least 50% of the main spike in each accession had reached this stage. To determine apical development (double ridge and awn primordium stages), four main stems per plot were sampled and dissected every three days to identify the stages of floret development (Nerson et al., 1980). The other stages (Anthesis, Tipping, Heading and Physiological Maturation) and developmental phases were established according to Alqudah and Schnurbursh (2014).

**Table 1:** External phenology of barley cultivars released at different decades in Brazil: thermal times (°Cd) from emergence (EM) to double ridge (DR), to the maximum number of spikelet primordia (MNP), to anthesis (ANT) and physiological maturity (PM) in 2011, 2012, and 2013 experiments.

Cultivar	DR	MNP	Ant	PM
FM 404	252 b^*^	622 b^*^	1281 a^*^	2013 ab^*^
FM 434	281 a	664 b	1220 b	1990 ab
BR 2	271 ab	633 b	1188 b	1892 b
BRS 195	277 ab	748 a	1335 a	2070 a
BRS Elis	268 ab	697 ab	1284 a	2006 ab

*Mean values followed by the same lowercase letters in a column do not differ statistically by Tukey’s test at 5%.

The developmental progress was characterized using thermal time units with a base temperature of 0°C (Cao and Moss, 1989). Weather data (temperature, photoperiod, rainfall, and humidity) were collected at Meteorological Station 83914, BDMEP/INMET) 100 m away from the experimental plots.

After emergence and before tillering, four subplots of one linear meter containing 60 plants were tagged in each plot. One linear meter was harvested when the plants reached the double ridge, awn primordium, anthesis, and maturity stages. A subsample of 10 plants was separated to evaluate the number of tillers, number of leaves, and green leaf area. The number of tillers per plant and leaf number on the main stem were measured in the stages of double ridge, MNP (maximum number primordium), and anthesis.

The leaf number on the main stem was linearly regressed against accumulated thermal time in the stages of DR and MNP, using the following equation:


Y=a+bx

Where “y” is the number of leaves, “a” is the number of leaves when thermal time is 0, “b” is the rate of production of leaves, and “x” is the accumulated thermal time from seedling emergence. The Phyllochron was estimated as the inverse of the corresponding slopes. Therefore, 1/b represented the phyllochron for the leaves in the stages of DR and MNP. Tiller mortality was calculated as the relative difference between tiller number at maturity and maximum number primordium (MNP).

The solar radiation intercepted by the crop was calculated as R*Int* (%)=100-(R*tran* x *RInc* ^-1^) x 100, where R*Int* is the proportion of solar radiation intercepted by crop, RInc is the incident radiation above the canopy, and Rtran is the transmitted radiation through the canopy and measured as the incident radiation below the last layer of green leaves. Rtran and RInc were measured twice weekly at midday under clear sky conditions, using a linear PAR/LAI Ceptometer (Accupar PAR-80, Decagon Devices Inc.)

The leaf area index (LAI) (leaf blade) was measured in the stages of DR, MNP, and ANT using the LI-COR LI-3000 area meter (LI-COR Inc, Lincoln, NE). **Table 2** summarizes all abbreviations used in the text.

**Table 2:** Abbreviations used in the text to designate each of the quoted traits.

Abbreviation	Trait
EM	Emergence
DR	Doble ridge
MNP	Maximum number of spikelet primordia
ANT	Anthesis
TS	Terminal spikelet
FLN	Final leaf number
LAI	Leaf area index
AP	Awn primordium
GF	Grain filling period
LS	Leaf and spikelet differentiation phase (°C d
SE	Stem elongation phase (°C d)
PM	Physiological maturity

## Results

### Duration of phenological phases

The cultivars under study showed differences in the duration of the total cycle, with cultivar BR 2 the shortest cycle (1892°C d) and cultivar BRS 195 the most extended (2070°C d) (**Table 1**).

The duration of the phase between emergence until anthesis (**Table 1**) was not significantly associated with the years of release (r^2^ = 0.37 n.s). Even so, breeding affected the proportion of DR-MNP and MNP-ANT periods to the total time to anthesis (**Table 3**). The MNP-ANT period was reduced from 1977, which means that this change was made in a shorter period of time. On the other hand, the DR-MNP period was increased in new cultivars (**Table 3**), from 2001 onwards.

**Table 3:** Duration of the different phenological phases of barley cultivars released at different decades in Brazil: thermal times (° Cd) from emergence to double ridge (EM-DR); DR to the maximum number of primordium (DR-MNP); MNP to Anthesis (MNP-ANT) and DR-MNP to MNP-ANT ratio in 2011, 2012 and 2013 experiments^(1)^.

Cultivar	EM-DR	DR-MNP	MNP-ANT	EM-MNP	DR-MNP to MNP-ANT/Ratio
FM 404 (1968)	252 b	370.8 b	659 a	622.46 b	0.57 c
FM 434 (1977)	281 a	383.2 b	556 b	664.40 b	0.69 abc
BR 2 (1989)	271 ab	361.7 b	555 b	632.83 b	0.65 bc
BRS 195 (2001)	277 ab	470.5 a	587 b	747.83 a	0.81 a
BRS Elis (2008)	268 ab	428.9 ab	587 b	697.16 ab	0.74 ab

(1) Averages followed by the same letter in the column do not differ statistically by Tukey's test at the 5% probability level.

(p<0.05) as tested by Tukey’s multiple range test.

**Figure 1:**
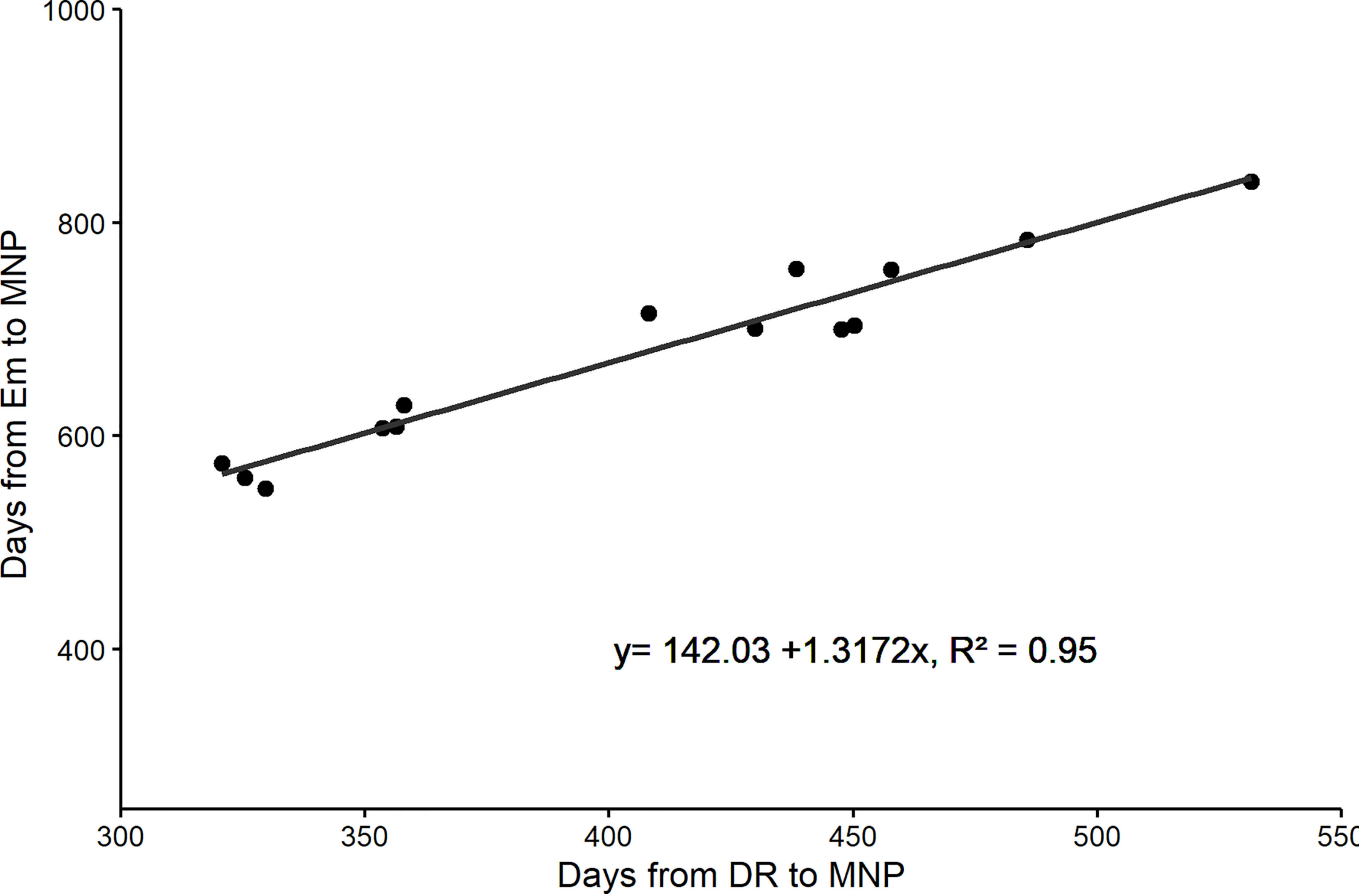
Relationship between days from emergence to Maximum number of spikelet primordia (MNP) and days from Doble ridge (DR) to MNP for five cultivars of barley released at different eras in Brazil (FM 404-released in 1968, FM 434-released in 1977, BR 2-released in 1989, BRS 195-released in 2001, and BRS Elis-released in 2008). Data correspond to the experiments carried out in 2011, 2012, and 2013.

In the modern cultivars, the duration of the EM-DR and DR-MNP sub-phases increased and consequently amplified the duration of the EM-MNP period, highly associated with the duration of DR-MNP phase (**Figure 1**), since there were no differences between cultivars in the duration of the sub-phase EM-DR (**Table 3**). Such contribution from genetic improvement affected the proportion of DR-MNP/MNP-ANT period concerning the total time to anthesis.

When the yields of the cultivars were evaluated as a function of the duration of the MNP-ANT period, no significant correlation was observed (y=-3.3294x +7271.3, r^2 =^ 0.041, n=15 p > 0.05). On the other hand, the duration of the DR-MNP period was significantly associated with the evolution of the grain yield of the cultivars (y= 9.4627x + 1497.6 r^2 =^ 0.499, n=15, p < 0.05), characterizing the importance of the period (DR-MNP) for grain yield evolution.

### Final leaf number and phyllochron

**Figure 2:**
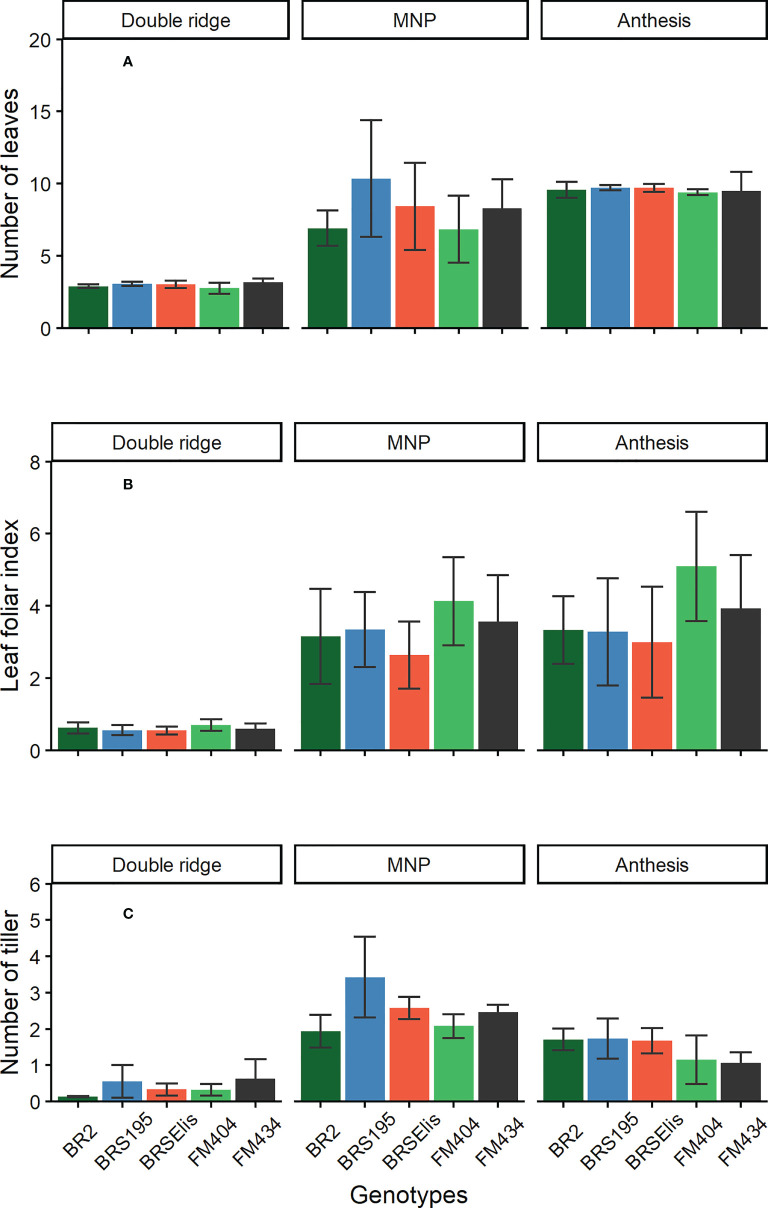
Number of leaves **(A)**, leaf foliar index **(B)** and number of tillers **(C)** in the main stem in different development stages of barley cultivars. MNP, Maximum number of spikelet primordia. Bars stand for the standard error (SE) of the means.

The final leaf number (FLN) per main stem evaluated in the DR was not different among the cultivars released during the analyzed period (**Figure 2A**). The same behavior was observed among the cultivars in the anthesis phase, and in the average of the three years evaluated, the FLN per plant was 9.4, 9.5, 9.6, 9.7, and 9.7 for the cultivars FM 404, FM 434, BR2, BRS 195 and BRS Elis, respectively. However, the number of leaves evaluated in the MNP stage was slightly higher in the newer cultivars (BRS 195 and BRS Elis). At this stage, the cultivar FM 404 showed the lowest number of leaves and BRS 195 the highest.

The phyllochron of the newer cultivars was smaller than the older cultivars. The phyllochron of new cultivars was 63°C per day per leaf (average of BRS 195 and BRS Elis) and 83°C per day per leaf for older cultivars (average of FM 404, FM 434, and BR 2) (**Table 4**). The relationship between phyllochron and the duration of the DR-MNP phase (**Table 3**) and phyllochron (**Table 4**) of the cultivars under study was significantly negative (y = -3.1983 x + 629.8, r^2 =^ 0.96, n= 5; p < 0.001). The duration between MNP-Anthesis did not show a significant relationship with the phyllochron.

**Table 4:** Phyllochron for five barley cultivars released at different eras in Brazil (FM 404; FM434, BR 2; BRS 195 and BRS Elis).

Cultivars	Phyllochron^*^	Linear equation	r^2^
	(°C per day per leaf)		
FM 404(1968)	86.2	y = 0.0116 x - 0.2776	0.90
FM 434(1977)	74.0	y = 0.0135 x - 0.6521	0.94
BR 2 (1989)	89.2	y = 0.0112 x - 0.1293	0.93
BRS 195 (2001)	59.2	y = 0.0169 x - 1.8211	0.80
BRS Elis (2008)	67.6	y = 0.0148 x - 1.1946	0.89

*Phyllochron was calculated as reciprocal of the slope pf a linear function between evolution of NFF and thermal time from emergence (base temperature of 0°C).

Linear equation and regression coefficient between the final number of leaves and thermal time (data represent the average of four repetitions, obtained in the DR and MNP stages in 2011, 2012, and 2013).

### Leaf area index and tillering

The Leaf area index (LAI) evaluation did not reveal differences between cultivars at the DR stage (**Figure 2B**). However, there were slight differences between the MNP and Anthesis stages. In general, in these stages (MNP and ANT), the oldest cultivars (FM 404 and 434) showed a slight superiority (**Figure 2B**) compared to the newer cultivars. On the other hand, for each specific cultivar, there were no marked differences in LAI between the stages of MNP and Anthesis despite the anthesis stage incorporating the leaf area loss due to tiller mortality in this period (MNP-anthesis) (**Figure 2B**). The IAF at the MNP stage was 2.6; 3.3; 3.1, 3.6, and 4.1, and at anthesis it was 3.0; 3.3; 3.3; 3.9, and 5.1 for cultivars BRS Elis, BRS 195, BR 2, FM 434 and FM 404, respectively for each stage.

The maximum number of tillers per plant was observed at the MNP stage where cultivar BRS 195, BRS Ellis, FM 434, FM 404, and BR 2 presented 3.5; 2.6; 2.4; 2.2, and 1.9 tillers per plant, respectively (**Figure 2C**). Modern cultivars (BRS 195 and BRS Elis) have more tillers than older cultivars. The final number of tiller (anthesis) was superior in modern cultivars (BR 2; BRS 195 and BRS Elis) and was related to the maximum number of tiller (y = -0.1415 x^2^ 1.429 x - 0.7616; R^2^ = 0.73, n = 15, p <0.01) evaluated at the MNP stage (data not shown). High tiller cultivars showed low tiller mortality rates (y = 0.0009x^2^ + 0.0385x +1.7488; R^2^ = 0.77, n = 15).

**Figure 3:**
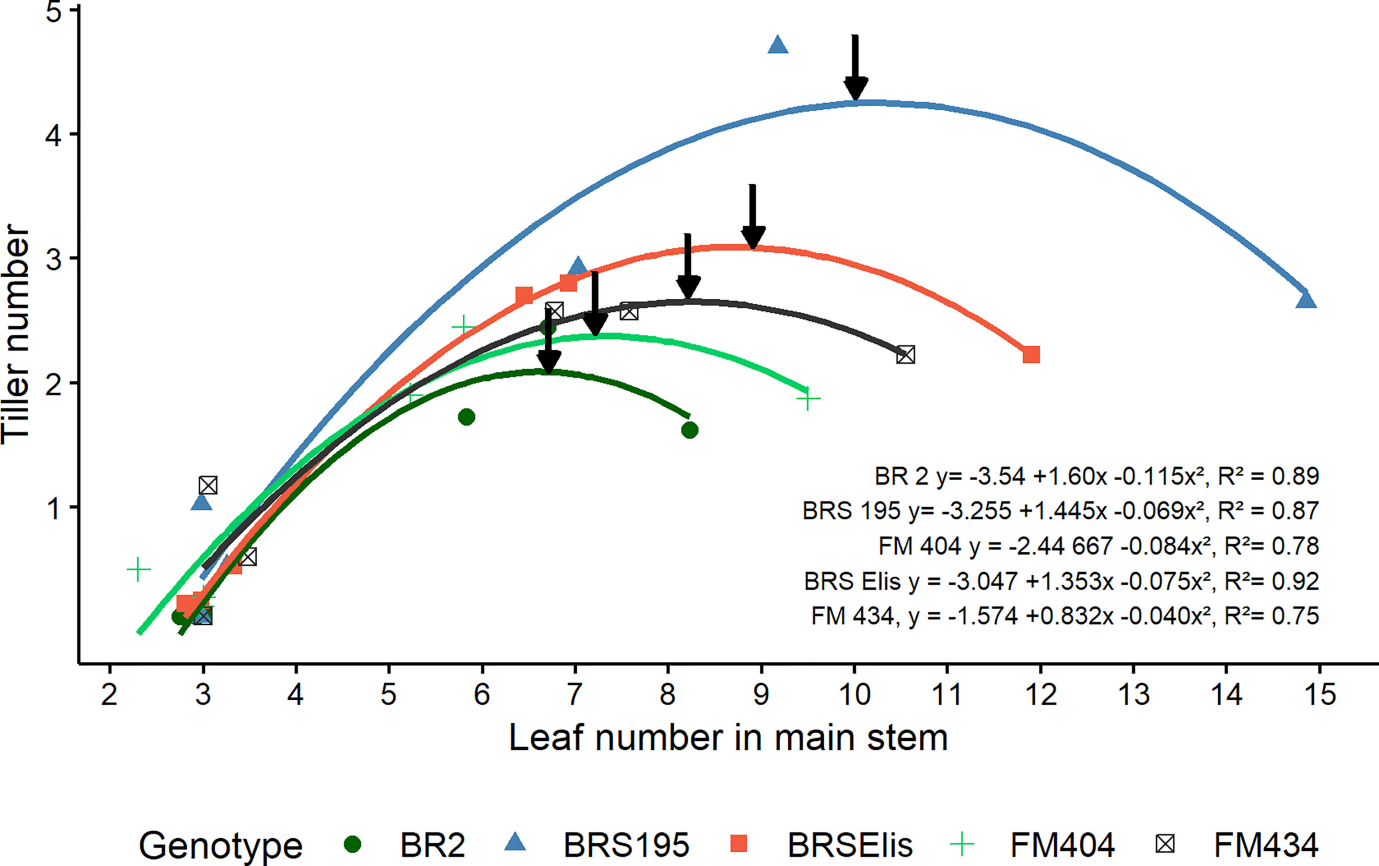
Dynamics of tillering as a function of leaf number in the main stem of barley cultivars released in different decades in Brazil. The arrows indicate the maximum number of tillers. The points represent the average values of 4 repetitions obtained in the Doble ridge (DR) and Maximum number of spikelet primordia (MNP) stages, in the years 2011, 2012, and 2013.

The relation between the number of tillers as a function of the number of leaves in the main stem assessed at the DR and MNP in the three years of study was best described by a quadratic function (**Figure 3**) for all cultivars. It was observed that the modern cultivars (BRS 195 and BRS Elis) presented the smallest Phyllochron (59.2 and 74.6), respectively. Modern cultivars showed faster leaves and tillers development, which allows an earlier canopy closure.

### Interception of radiation

**Figure 4:**
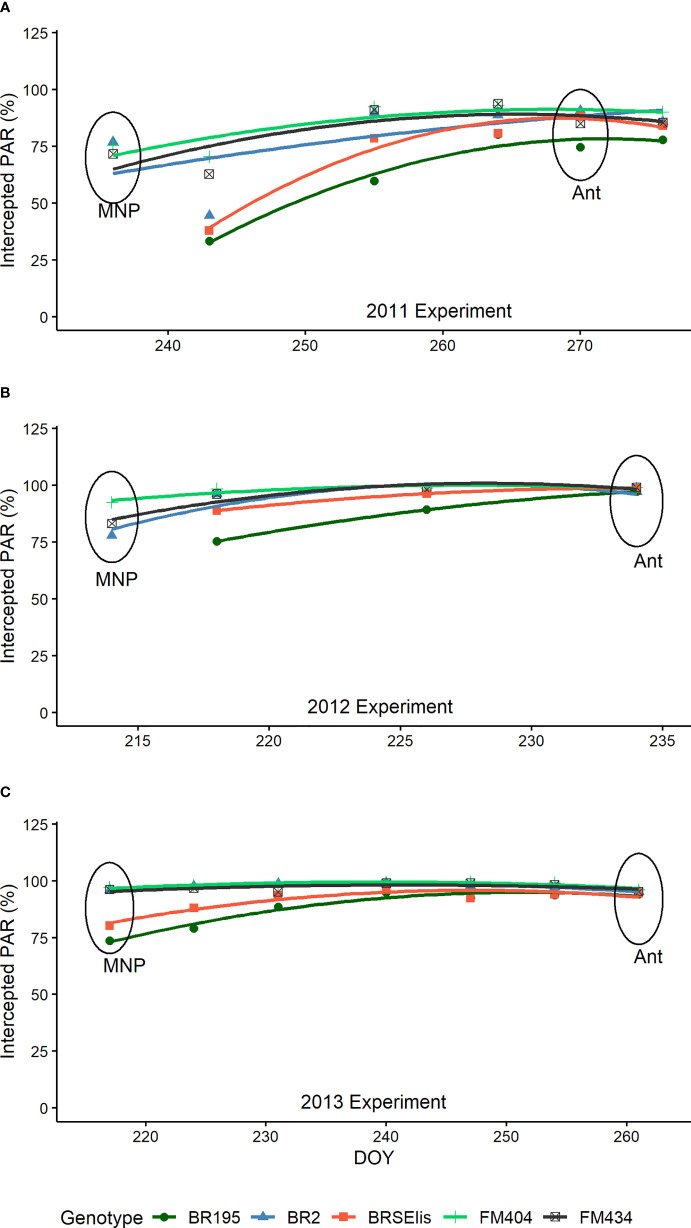
Photosynthetically active radiation (PAR) intercepted by barley cultivars released in different decades in Brazil in 2011, 2012, and 2013 experiments **(A–C)**. The circles indicate the position of the phenological stages evaluated. MNP, Maximum number of spikelet primordia; Ant, Anthesis.

Regarding radiation interception, it was observed that for modern cultivars (BRS 195 and BRS Elis) in the MNP stage, radiation interception was significantly lower than the other cultivars in the three years of study (**Figure 4**). At the anthesis stage, except for the cultivar BRS 195 in 2011, the radiation interception was maximum and showed no differences between the cultivars studied.

### Developmental stages

**Figure 5:**
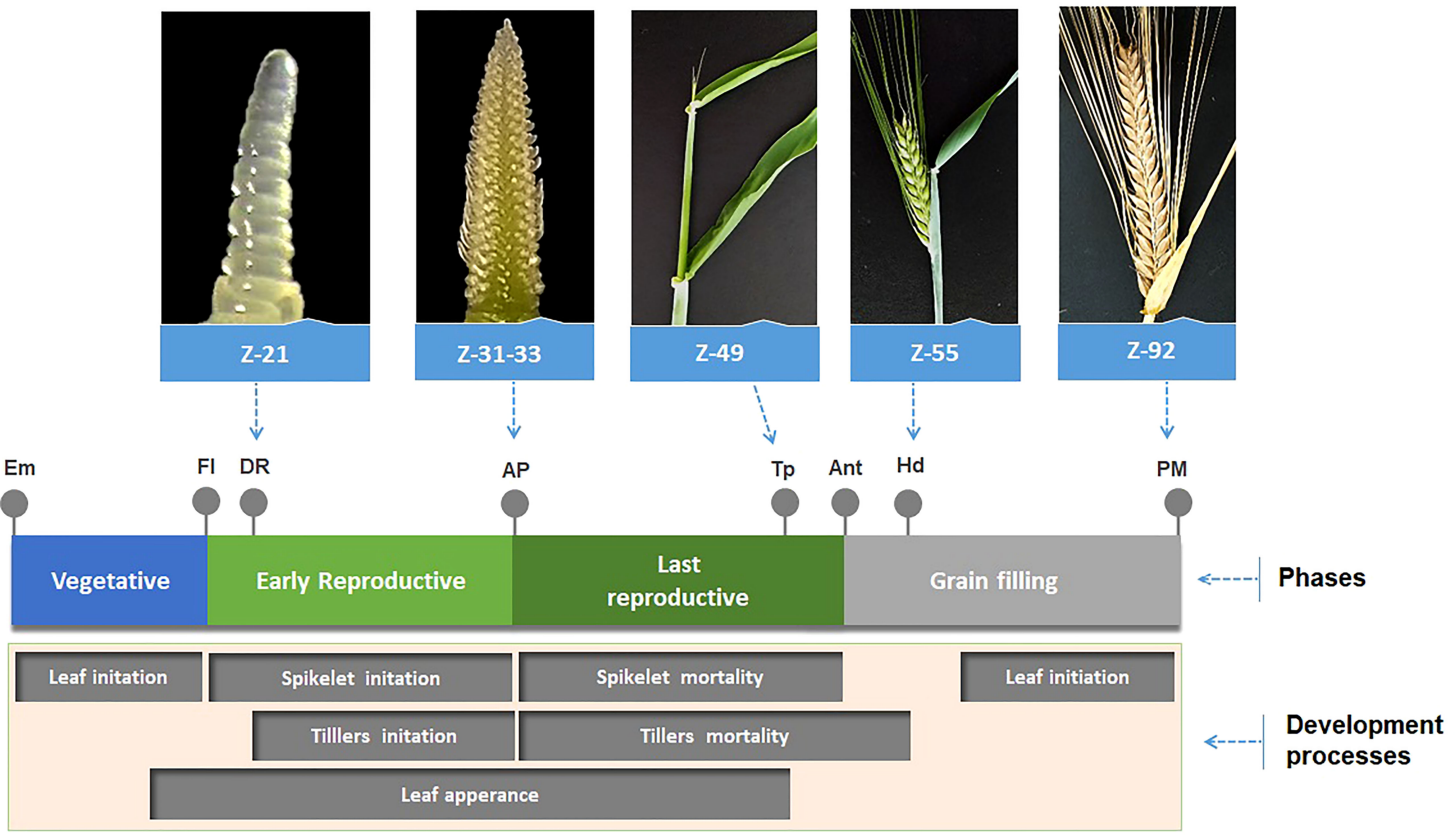
Schematic diagram of barley development showing the different stages (Em, Emergence; FI, Floral Initiation; DR, Double Ridge; AP, Awn Primordium (or MNP, Maximum number of spikelet primordia); Tp, Tipping; Ant, Anthesis; Hd, Heading; PM, Physiological Maturity; Z(n), Zadoks scale; phases and development processes.

**Figure 5** is a development diagram showing the different stages of barley development for a better understanding of each stage discussed in this investigation.

## Discussion

The results of this work demonstrate that, although there was no significant association in the cultivars cycle length (EM-ANT) with the year of release, the genetic improvement affected the proportion of DR-MNP/MNP-ANT period to the total time to anthesis (**Table 3**). The higher proportion of the DR-MNP period to the MNP-ANT period in modern cultivars than in old ones, may have come from breeding efforts to reduce plant height and maintain the time to anthesis. Such condition could have sustained a greater production of reserves used in the production of primordial spikelet. The greater correlation observed between the duration of this period (DR-MNP) and the grain yield presented by the cultivars, reflects the importance of the first reproductive phase (DR-MNP) for yield gains. Such results could help breeders to focus on this critical period of development to maximize grain production in barley.

Results presented by Miralles et al. (2000), despite having studied a single cultivar (one genetic background) of barley, in a way corroborate our results. In that study, they observed that in two-row barley, the elongation of the MNP-ANT period did not have the same impact on the increase in the number of fertile flowers per spike caused in wheat. In barley, these results already pointed out that this period (MNP-ANT) is not as limiting in barley as it is in wheat. Therefore, it is to be expected that the DR-MNP phase in two-row barley that has an undetermined ear may have been chosen to advance the evolution of genetic material to maximize grain production.

Abeledo et al. (2003) observed different results in barley cultivars releases between 1944 and 1998 in Argentina, where a higher contribution of the total time (EM-ANT) to the elongation period of the stem (MNP-ANT) was observed. On the other hand, in the same study, when the cultivar Beka was excluded from the list of cultivars participating in the regression analysis, they observed results similar to present study. It was also observed a significant association between the release years and the period between emergence and MNP, including the DR-MNP period. Finally, the increase in the duration of the DR-MNP phase in the newer cultivars at the expense of the MNP-ANT phase duration, without a consistent change in the anthesis time (EM-ANT), might be responsible for the increase in the number of grains/m^2^ and yield verified by Rodrigues et al. (2020).

The reflection of the increase in the duration of the final reproductive phase (MNP-ANT) in the number of grains per m^2^ in two-row barley is smaller than wheat with multi-floral spikelets. The number of grains m^-2^ in wheat is expected to depend more on the number of viable flowers within the spikelets. Whereas in two-row barley, the number of grains m^-2^ is expected to rely more on the number of spikelet primordia determined by the duration of the DR-MNP phase (Miralles et al., 2000).

Alquhad and Schnurbusch (2014) demonstrated that the sub-phase between MNP and the tipping is critical for increased barley yield potential through increased development and survival of spikelets. However, the materials we studied indicate that this sub-phase was not the most important to support the grain yield potential. In the case of Brazilian genetic material, the generation of spikelet primordia supported by the sub-phase between DR-MNP seems to be the path chosen for the advancement of yield potential rather than the reduction of spikelet abortion initiated shortly after MNP until tipping.

In this condition, the increase in the period between DR-MNP (i.e., the definition of the number of spikelets and initiation of the tillers) may have favored an increase in the number of spikes and grain m^-2^. The reduction in the period MNP-ANT, where spike and stem growth are competing for reserve, and the progress in reducing plant height and lodging of modern cultivars (i.e., shortening of internodes) (Rodrigues et al., 2020) may have favored spike growth and reduced the mortality of the tillers.

The increase in the length of the DR-MNP period in the genetic material studied may have favored a larger number of spikelets since the differentiation of spikelets in the barley ear is indeterminate. Therefore, as in two-row barley, only the central spikelet is fertile in each rachis internode, and a larger number of spikelets can provide a higher proportion of heavier grains (greater ovary) in total grains m^-2^ (Calderini et al., 2001). In this context, shortening the MNP-ANT period as a function of the DR-MNP elongation may not negatively reflect the reserve competition within the spikelet since barley of two rows has only one flower primordium per spikelet (Arisnabarreta and Miralles, 2006). Thus allowing a relative partition of the development time for the DR-MNP period without compromising the duration of the total period (DR-ANT). The elongation of the DR-MNP period at the expense of the later phase may be an interesting strategy to improve grain yield in barley.

Regarding the FLN on the main stem, there was no change due to the improvement of barley malting in Brazil in the analyzed period (1968-2008). However, the FLN in the MNP stage was slightly higher in the newer cultivars (BRS 195 and Elis), which could support a higher number of tillers in the MNP stage (**Figure 2A**). As there was no difference in the FLN between the cultivars in the anthesis stage, the biggest difference in the FLN observed in the newer cultivars (BRS 195 and BRS Elis) in the MNP stage could be attributed to the faster establishment of the leaves, favoring a better support condition for a greater number of tillers at the MNP stage (**Figure 2C**).

The reduction of phyllocron in modern cultivars allowed the occurrence of faster leaves, contributing to the early canopy closure and tiller viability. Likewise, increasing the duration of the DR-MNP phase at the expense of reducing the subsequent phase (MNP-ANT) in modern cultivars improves the canopy structure of the crop before the MNP phase. Also, considering that tillering is a photomorphogenic process, it would be possible to speculate that the decrease in the value of the phyllochron presented by modern cultivars could be the cause of the increase in the tillering rate (**Figure 2C**).

Regarding the leaf area index (LAI), it was observed that modern cultivars presented the lowest indexes (**Figure 2C**) compared to the oldest cultivars in the MNP and ANT stages. Such behavior may have enabled a greater radiation availability, mainly at the MNP stage, favoring tillering and its viability. The highest LAI at the anthesis stage, observed in the oldest cultivars (FM 404 and FM 434) compared to modern cultivars, may be due to the larger leaf area since the number of leaves was not different between cultivars and the number of tillers was inferior in these cultivars.

The LAI and radiation interception of cultivars in the MNP-ANT period (**Figure 4**) allowed us to observe that the improvement in the analyzed period changed the leaf behavior (foliar architecture). The lower interception of radiation at the MNP stage (**Figure 4**) compared to the anthesis stage, without reducing LAI in modern cultivars (**Figure 2C**), can be attributed to the more vertical leaf inclination of these cultivars (erectophile canopy) favoring the penetration of PAR into the crop canopy. As tillering is a photomorphogenic process (Casal, 1988; Lauer and Simmons, 1989), greater radiation availability may have favored the induction of a greater number of tillers and better survival.

From that stage (MNP), the leaves of modern cultivars were tilted, intercepting more radiation (planophile canopy) at the anthesis stage. Similar results were observed in Argentina (Abeledo et al., 2004). The breeding modified the intercepted radiation accumulated during the MNP-ANT period when the number of viable tillers was established. In Brazil, the genetic improvement of barley had improved tillering and its viability by modifying the leaf architecture and radiation interception

Modern cultivars (BRS 195 and BRS Elis) have more tillers than older cultivars, which is associated with an increased tillering rate. The final number of tillers was related to the maximum number of tillers, and the high tiller cultivars showed low tiller mortality rates. Thus, the improvement of barley in Brazil in the analyzed period increased the tillering through the increase in the tillering rate and the low tiller mortality rate. It also was observed that the modern cultivars reached the maximum number of tillers with a larger number of leaves (**Figure 3**). This larger number of leaves, at an earlier period, may have sustained the largest number of viable tillers (number of ears) and explained the genetic advance of barley production in the analyzed period, since the tillering is one of the most critical components used by cereals to increase grain yield (Sreenivasulu and Schnurbusch, 2012; Xie et al., 2016). On the other hand, the increase in the initial reproductive period (DR-MNP) as a result of the reduction in the stem elongation period (MNP-ANT) by the effort of barley improvement, could have reduced the competitiveness of the stem by reserve, favoring the viability of tillers, since there is a positive association between the elongation of the main stem and the death of late tillers (Hay, 1986). This potential impact on reducing the elongation period of the stem could have reduced the pressure on the mortality of the tillers, thus improving the number of tillers and spikes m^-2^. Riggst et al. (1981) found in modern barley cultivars high rates of production and survival of tillers.

In conclusion, barley breeding in Brazil has: (1) increased tillering and the number of viable tillers (number of ears); (2) increased the proportion of the DR-MNP/MNP-ANT period to the total time until anthesis by reducing the duration of the MNP-ANT period, reducing the competition between stem and tiller growth; (3) did not change the total number of leaves on the main stem, but caused an increase in the number of leaves during early development, increasing the number of viable tillers (number of ears); (4) reduced LAI and modified the leaf architecture of modern barley, making the leaf inclination more vertical (erectophilic canopy) allowing the penetration of photosynthetically active radiation into the crop canopy.

## Data availability statement

The raw data supporting the conclusions of this article will be made available by the authors, without undue reservation.

## Author contributions

Authors OR and EM: conceptualization, methodology, investigation, data curation, funding acquisition. OR, EC, JM, and SS: formal analysis. OR and EM wrote the manuscript. OR and JM: editing and revision. OR, SS, and EC: making of graphs, and figures. All authors contributed to the article and approved the submitted version.

## Funding

We are grateful for the financial and infrastructure support provided by the Brazilian Agricultural Research Corporation, Embrapa Wheat.

## Acknowledgments

We thank Eduardo Beche for the English language revision.

## Conflict of interest

Authors OR, EM, and EC were employed by Brazilian Agricultural Research Corporation.

The remaining authors declare that the research was conducted in the absence of any commercial or financial relationships that could be construed as a potential conflict of interest. The authors declare that this study received funding from Brazilian Agricultural Research Corporation, Embrapa Wheat.

The funder was not involved in the study design, collection, analysis, interpretation of data, the writing of this article, or the decision to submit it for publication.

## Publisher’s note

All claims expressed in this article are solely those of the authors and do not necessarily represent those of their affiliated organizations, or those of the publisher, the editors and the reviewers. Any product that may be evaluated in this article, or claim that may be made by its manufacturer, is not guaranteed or endorsed by the publisher.
